# Postcopulatory Sexual Selection Is Associated with Reduced Variation in Sperm Morphology

**DOI:** 10.1371/journal.pone.0000413

**Published:** 2007-05-02

**Authors:** Sara Calhim, Simone Immler, Tim R. Birkhead

**Affiliations:** Department of Animal and Plant Sciences, University of Sheffield, Sheffield, United Kingdom; University of Oxford, United Kingdom

## Abstract

**Background:**

The evolutionary role of postcopulatory sexual selection in shaping male reproductive traits, including sperm morphology, is well documented in several taxa. However, previous studies have focused almost exclusively on the influence of sperm competition on variation among species. In this study we tested the hypothesis that intraspecific variation in sperm morphology is driven by the level of postcopulatory sexual selection in passerine birds.

**Methodology/Findings:**

Using two proxy measures of sperm competition level, (i) relative testes size and (ii) extrapair paternity level, we found strong evidence that intermale variation in sperm morphology is negatively associated with the degree of postcopulatory sexual selection, independently of phylogeny.

**Conclusions/Significance:**

Our results show that the role of postcopulatory sexual selection in the evolution of sperm morphology extends to an intraspecific level, reducing the variation towards what might be a species-specific ‘optimum’ sperm phenotype. This finding suggests that while postcopulatory selection is generally directional (e.g., favouring longer sperm) across avian species, it also acts as a stabilising evolutionary force within species under intense selection, resulting in reduced variation in sperm morphology traits. We discuss some potential evolutionary mechanisms for this pattern.

## Introduction

The evolutionary role of postcopulatory sexual selection in shaping male reproductive morphology, physiology and behaviour is well documented in several taxa [1]–[4]. In particular, postcopulatory sexual selection has been shown to affect several ejaculate traits, including sperm morphology [5], [6].

Sperm are amongst the most variable cells across animal taxa [7], and this variation can be examined at different taxonomic levels, from phyla to species, individuals and ejaculates [8]. To date most studies have focussed on interspecific differences in sperm morphology [9]–[22], probably because variation between species is generally assumed to be greater than within species [e.g. 23]–[25]. There is evidence that, as with sexual selection in general, postcopulatory sexual selection is a directional evolutionary force, favouring longer or more elaborate sperm in certain taxa, including birds [e.g. 9]–[11], [13], [15], [16], [21]. In contrast, the effect of postcopulatory sexual selection on the variation in sperm morphology between individual males is largely unknown.

Theoretical models of sperm size evolution [26]–[28] predict that under diploid control (i.e. male genotype), certain sperm trait optima might exist at given levels of sperm competition. Diploid control models predict that under intense postcopulatory sexual selection males are selected to produce sperm whose morphology matches these optima, whereas males under less intense selection are not [27]. In other words, when postcopulatory sexual selection is relaxed, we predict greater intermale variation in sperm traits than when postcopulatory sexual selection is intense. The few available data are consistent with this prediction. In the hopping mouse (*Notomyx alexis*) for example, intermale variation in sperm head morphology is greater than that of the closely related species, *Pseudomys australis* [29]. Across primates, Harcourt [30] provided data suggesting that intermale variation in sperm length and mating system were associated, but did not perform any formal analyses. In both studies, the lowest variation in sperm morphology was observed in the taxa where females are polyandrous and/or males have relatively large testes, and therefore under intense postcopulatory sexual selection [29], [30]. Among birds, Birkhead et al. [25], [31] also suggested that the high degree of intermale variation in sperm design in the zebra finch (*Taeniopygia guttata*) and Eurasian bullfinch (*Pyrrhula pyrrhula*) may be the result of relaxed postcopulatory sexual selection.

Here we test the hypothesis [25] that intermale variation in sperm morphology is negatively associated with the level of postcopulatory sexual selection. This study is the first to formally test the effect of selection acting on *intraspecific variation* using a phylogenetic framework. Using data for 18 species of passerine bird, two indices of intraspecific variation in sperm morphology (sperm length and sperm design; see Methods), and two indices of postcopulatory sexual selection (relative testes size and extrapair paternity level), we found clear support for this hypothesis.

## Results

**Figure 1:**
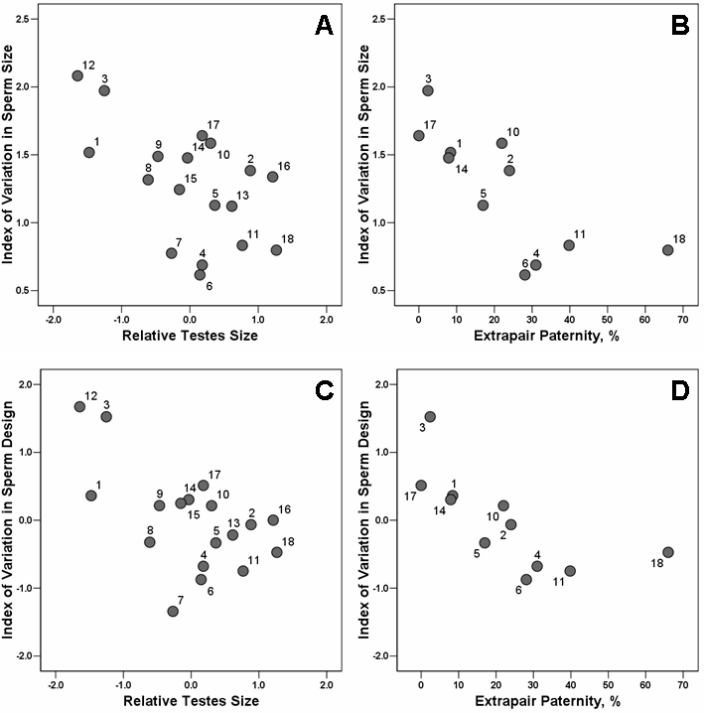
Negative relationships between the degree of intermale variation in sperm morphology and the level of postcopulatory sexual selection (see Table 1). (A) Intermale variation in sperm size is negatively associated with relative testes size (*p* = 0.003, *n* = 18). (B) Intermale variation in sperm length is negatively associated with levels of extrapair paternity (*p*<0.001, *n* = 11). (C) Intermale variation in sperm design is negatively associated with relative testes size (*p* = 0.004, *n* = 18). (D) Intermale variation in sperm design is negatively associated with levels of extrapair paternity (*p* = 0.006, *n* = 11). All analyses were performed controlling for phylogeny and sample size using Generalised Least-Squares Models and transformed variables. Relative testes sizes refer to residuals from a regression of log-transformed combined testes mass on body mass. Extrapair paternity levels refer to percent of offspring not sired by the alpha male. See Methods for more details. Species list (*n* values refer to the number of individual males sampled): 1, *Ficedula hypoleuca* (*n* = 40); 2, *Prunella modularis* (*n* = 56); 3, *Taeniopygia guttata* (*n* = 51); 4, *Quelea* *quelea* (*n* = 236); 5, *Fringilla coelebs* (*n* = 47); 6, *Agelaius phoeniceus* (*n* = 38); 7, *Seiurus aurocapillus* (*n* = 10); 8, *Mniotilta varia* (*n* = 10); 9, *Protonotaria citrea* (*n* = 10); 10, *Geothlypis trichas* (*n* = 10); 11, *Setophaga ruticilla* (*n* = 10); 12, *Phyrrula phyrrula* (*n* = 19); 13, *Carduelis flammea* (*n* = 12); 14, *Acrocephalus shoenobaenus* (*n* = 15); 15, *Acrocephalus scirpaceus* (*n* = 14); 16, *Sylvia atricapilla* (*n* = 10); 17, *Zosterops lateralis* (*n* = 42); 18, *Malurus cyaneus* (*n* = 59).

For both indices of intraspecific variation in sperm morphology ([i] index of variation in sperm size and [ii] index of variation in sperm design [see Methods]) and for both estimates of the intensity of postcopulatory sexual selection ([i] relative testes size and [ii] percent of extrapair offspring [see Methods]), the degree of variation decreased with postcopulatory sexual selection intensity (Figure 1, Table 1).

**Table 1:** Summary of the Results.

Index of Sperm Variation	Index of Postcopulatory Sexual Selection	Slope±s.e.	t	p	R^2^
Sperm Size	Relative Testes Size				
	Testes mass	−0.30±0.08	−3.54	**0.003**	0.46
	Body mass	0.17±0.18	0.90	0.381	
	Extrapair Paternity	−0.02±0.01	−4.89	**<0.001**	0.73
Sperm Design	Relative Testes Size				
	Testes mass	−0.46±0.14	−3.30	**0.004**	0.42
	Body mass	0.13±0.30	0.42	0.679	
	Extrapair Paternity	−0.02±0.01	−3.52	**0.006**	0.58

Generalised Least-squares (multiple) regression analyses controlling for phylogeny and sample size.

Index of Variation in Sperm Size = coefficient of variation (CV) in sperm total length.

Index of Variation in Sperm Design = scores of the first principal component (PC1) from a Principal Component Analysis of CVs of three independent sperm components: head, flagellum and midpiece lengths; PC1 explained 85% of the interspecific differences in CVs.

All the relationships above were independent of: (a) phylogeny: the fitted models did not differ significantly from equivalent models where the λ estimate was set as 0 (Likelihood Ratio Tests: P>0.99; see Ref. 21 and 76); and (b) sample size (GLS: sample size term, P>0.15).

All analyses conducted on transformed variables. See Methods for more details.

## Discussion

### Postcopulatory Sexual Selection: directional or stabilising?

Most sexually selected traits are under directional selection [32]. Very few studies however, have tested whether sexual selection affects the variance in these traits. In one such study, the degree of variation in a precopulatory sexually selected male trait (tail length) was found to be greater than other traits (e.g. wing and tarsus length) in the same species [33]. A more apposite comparison in the same study showed that intraspecific variation in tail length was much greater in species where tail length was under sexual selection than in species where tail length was not [33]. This difference is consistent with theory since sexually selected traits such as tail length are thought to be condition-dependent [e.g. 34].

In contrast to precopulatory sexually selected traits traits, there is almost no evidence that sperm morphology traits are influenced by environmental factors or are condition dependent [e.g. 35], [25]. Instead, sperm morphology traits show high heritabilities [36], [37], [25]. In the absence of any condition-dependence, theory predicts that selection would act to decrease variability within a species [e.g. 38]. We can therefore predict that postcopulatory evolutionary pressures in the evolution of sperm morphology which are directional across species [e.g. 11], could also constitute a stabilizing force at an intraspecific level. In other words, intense postcopulatory selection will act to decrease variation in sperm morphology within a species towards what might be an ‘optimum’ morphology.

In the following section we consider three possible evolutionary mechanisms that might favour an ‘optimum’ sperm morphology under strong postcopulatory sexual selection, through sperm competition [39] and/or cryptic female choice [40].

### Evolutionary Mechanisms for an Optimum Sperm Morphology

#### (a) Optimum Sperm Design and Sperm Competition

Under intense sperm competition, a more competitive ejaculate will always be favoured and an optimum sperm design might be linked to maximising sperm function (e.g. velocity, longevity). The size of two particular sperm components, (i) the flagellum (the sperm's ‘motor’), and (ii) the midpiece (the sperm's ‘powerhouse’) have been theoretically linked with sperm function [41], [42]. However, there is still no conclusive data on the relationship between sperm function and sperm morphology. In birds, the midpiece (and particularly mitochondrial function within it) has been shown to be positively linked with sperm motility [43], whereas in a mammal, midpiece size and sperm motility were negatively related [44].

#### (b) Optimum Sperm Size and Female Cryptic Choice

From a male's perspective, success in postcopulatory competition depends on achieving a balance in the theoretical trade-off between sperm size and numbers [26]–[28]. However, sperm competition does not act in isolation, and high levels of female polyandry also provide the opportunity for cryptic female choice and for females to be selective in the sperm they store and utilise. It is well established that in birds sperm selection occurs in the vagina soon after insemination, with only a few percent of inseminated sperm being retained by the female [45], [46]. There may also be selection at the level of the sperm storage structures since the length of sperm and length of the sperm storage structures positively covary across species [e.g. 47], [9], [11]. Similar patterns have been reported in several other taxa, and have been interpreted as an example of male-female coevolution, possibly mediated by sexual conflict over fertilisation [48]–[52]. In short, an optimum sperm length in birds might reflect a (temporary) resolution of the evolutionary arms-race with female sperm storage tubule length, which is the major force behind the interspecific pattern of positive directional selection in sperm length in birds [e.g. 9], [11].

#### (c) Genetic Factors

Two types of genetic factors may influence variation in sperm morphology and account for the pattern observed. First, negative genetic correlations between different sperm components may constrain sperm design and reduce the likelihood of achieving an ‘optimum’ design, especially when postcopulatory sexual selection is relaxed [25]. Second, if sperm phenotype is under diploid control (i.e. by the male genotype), certain sperm trait optima can be predicted at different levels of sperm competition [27]. However, under pure haploid control (i.e. individual sperm genotype) these optima break down, probably due to intra-ejaculate conflict [28]. Although it has not yet been modelled, we can speculate that the competition between males would be greater than the competition within ejaculates, if both diploid and haploid control exist, and are in conflict. Consequently, species under intense sperm competition would follow the diploid control pattern, resulting in a particular sperm trait optimum being selected. In contrast, intermale variability in sperm phenotype could persist in species with low levels of postcopulatory selection, as the result of unresolved diploid-haploid conflicts over the control of sperm phenotype [27], [28].

### Future Directions and Conclusions

While our results are consistent with the idea that sperm competition and/or cryptic female choice account for the degree of intermale variation in sperm morphology, the relative importance of these two processes remains to be established. If cryptic female choice is important, we might predict the variation in the dimensions of female sperm storage tubules will be less in species with high levels of female polyandry. Another prediction is that in artificial selection experiments in which the degree of female polyandry is increased, as in some studies of *Drosophila*, variation in sperm design would decrease, whereas under reduced polyandry the reverse would be true. Although such selection studies have been conducted [e.g. 53], so far researchers have focussed on mean male traits (e.g. sperm length) rather than the variance in these traits [e.g. 54]–[56].

In conclusion, this is the first study to explicitly test the role of post-copulation sexual selection as an evolutionary force acting on *intraspecific variation* in sperm morphology in a comparative framework. The fact that both intermale sperm size and design variability decrease with the level of postcopulatory sexual selection suggests that the latter is a strong stabilizing force in the evolution of avian sperm morphology. This is consistent with theoretical predictions for the effect of selection on variability of condition-independent traits [38], [34] and/or diploid control of sperm morphology [40]–[42]. Postcopulatory sexual selection therefore appears to have two types of evolutionary effects on avian sperm morphology: (i) a directional and positive effect on sperm size across taxa, where more promiscuous species generally have longer sperm [e.g. 9], [11]; and, subsequently, (ii) a stabilising effect resulting in a reduction in the variation in sperm design between males. It remains to be established precisely which factors drive this striking pattern.

## Material and Methods

### Sperm Morphology

We investigated intraspecific variation in sperm morphology in 18 species of passerine bird. Two methods were used to obtain sperm samples for morphometric analysis: (i) from the faeces of males in reproductive condition [57]; (ii) from the seminal glomera of dissected males in reproductive condition found dead or collected under licence. Samples were fixed in 5% formalin solution. Sperm morphometric data were obtained using digital image analysis software (Leica IM50 Image manager) and pictures taken using light microscopy. Five sperm per male were measured, since previous studies have shown that, in most instances, a sample of five sperm is representative [25], [21]. The following four sperm morphometric traits were measured (to the nearest 0.1μm): (i) sperm total length, (ii) head length, (iii) flagellum length, and (iv) straight midpiece length, hereafter referred to as midpiece length [see Ref. 25 for more details]. Repeatability of measurements was very high (several sperm traits repeatedly measured across different species; *r*_I_ range 0.90 to 0.99 [58]).

### Intraspecific Variation in Sperm Morphology

To date, most studies of sperm morphology have focused on total sperm length [6]. However, sperm morphology can also be assessed as overall sperm design, measured as the size of the different sperm components. We therefore used two indices of intraspecific variation in sperm morphology: (i) an index of variation in sperm size (log-transformed coefficient of variation, CV, in total length), and (ii) an index of variation in sperm design. For the latter, we conducted a Principal Component Analysis (PCA) on the log-transformed CV estimates for three independent measures of sperm morphology (head, flagellum and midpiece lengths). The scores of the first principal component, which explained 85% of the variation in the data, were used in subsequent analysis as the index of variation in sperm design. Head length, flagellum length and midpiece length were considered independent measures of sperm morphology as each can vary independently from any of the other two (pers. obs.; see Fig. 1 in Ref. 25). In addition, sperm size comprises the combined length of only two of the aforementioned sperm components–head and flagellum lengths–which can also differ in relative proportion across individuals with same total sperm length (pers. obs.; see Fig. 1 in Ref. 25). Although none of the four sperm traits measured was used in both indices, sperm trait sizes are intrinsically associated. Nonetheless, the two indices are not interchangeable: low variation in size does not preclude high variation in design, as two males may have sperm of very similar total length but differ markedly in the relative size of the individual sperm components (pers. obs.; see, for example, Fig. 1 in Ref. 25). Therefore, the two indices of variation might reflect different aspects of sperm morphology evolution. We therefore consider these separately.

Measures of variation in themselves are strongly influenced by differences between trait means and size of the sample [59]. Using the coefficient of variation (CV) as a measure of variability controls for differences in trait size across species, but controlling for different sample sizes, and for small samples in particular, is more difficult. Applying Haldane's small sample correction is not appropriate because the expected error is always greater than the correction itself [59]. Therefore, to determine the appropriate number of individual males to be sampled we undertook sampling simulations using two species which represent extremes of postcopulatory sexual selection: the zebra finch and the superb fairy-wren (*Malurus cyaneus*), for low and high levels respectively [60], [61]. A mean estimate of CV in sperm total length across repeats was calculated at each *n* (see Methods S1, Figure S1). The results from these sampling simulations suggest that in order to accurately assess variation, the minimum adequate *n* is 10 males, for all but the most extreme cases. We were able to obtain data for at least *n* = 30 males (the most conservative sample size, see Figure S1) for 8 out of 18 species sampled. To further control for potential effects of sample size differences between species, sample size was included in every model, although this term was later removed in all cases as it failed to have a significant effect.

### Measures of Postcopulatory Sexual Selection

Relative testes size (testes size controlled for body size) and level of extrapair paternity (EPP) are the two most widely used indices of the intensity of sperm competition [e.g. 9], [61]–[68]. At the present, it is not clear which index is the most appropriate, as not all data available for either one are reliable [69], [70]. Although the two indices are likely to be positively associated [63]; [but see 69], each may be affected by factors other than postcopulatory sexual selection. For example, relatively large testes can also be a response to sperm depletion risk [e.g. 71] and low EPP values can be found despite high incidence of extra-pair copulation [e.g. 72]. We therefore used both indices in the current analysis. Data on EPP levels, measured as the percentage of offspring not sired by the (alpha) social male, were obtained from the literature (see Supporting Information). Combined testes mass (CTM) and body mass (BM) data were also obtained from published datasets (see Supporting Information). Although there was no relationship between logarithmically transformed CTM and BM (Linear regression, *p* = 0.07), the potential confound of allometry in testes mass and body mass across the *n* = 18 species sampled was controlled for by incorporating both CTM and BM as predictors in the model. The term BM never showed a significant effect but was retained (see Table 1).

### Statistical Analysis

All the statistical and simulation analyses were conducted using R v.2.3.1 [73]. All variables were transformed prior to analysis (arc-sin transformation of extrapair paternity levels and natural logarithms for all others). In order to account for non-independence of points due to shared ancestry [74], [75], a generalised least-squares (GLS) approach in a phylogenetic framework was used [76]. The GLS methods allows the estimation of λ, a phylogenetic scaling parameter between zero (no phylogenetic effect) and one (phylogeny completely explains the pattern), which is then incorporated in the model (see Ref. 21, for further details on the GSL method and phylogeny used in the current study).

## Supporting Information

Methods S1 Adequate Sample Size Simulations and Sources of Data
(0.03 MB DOC)

Figure S1 Bootstrapped estimate of the intraspecific coefficient of variation (CV) in sperm tota length (solid lines) against sample size, in species under (i) low or (ii) high sperm competition. The dashed lines correspond to the CV estimate using the complete sample for each species. Note that the *n* at which the solid lines level off is different in the two cases.
(0.04 MB TIF)
