# Supplementary material for: Postcopulatory Sexual Selection Is Associated with Reduced Variation in Sperm Morphology
Source: PLoS One. 2007 May 2;2(5):e413. doi: 10.1371/journal.pone.0000413 (PMC1855076; doi:10.1371/journal.pone.0000413)
Supplement: Methods S1 — Adequate Sample Size Simulations and Sources of Data (0.03 MB DOC) [file pone.0000413.s001.doc]

**Simulations for Adequate Sample Size**

Data from two species were used: (i) low sperm competition (*Taeniopygia guttata*), and (ii) high sperm competition (*Malurus cyaneus*). The coefficient of variation (CV) in sperm length was estimated at increasing sample sizes (minimum *n* = 5, *n* = 1 increments; 10000 repeats; sampling without replacement option). Sampling simulations were conducted using R v.2.3.1 (http://www.R-project.org). The resulting bootstrapped estimate of CV was then computed at every *n* for each species. See Figure S1.

**Sources of Data and References**

Combined testes mass and body mass data were obtained from published datasets (Calhim & Birkhead 2007, and references therein). Percent of extrapair paternity data were obtained from Griffith et al. (2002) for all species but *Geothlypis trichas* (Thusius et al. 2001) and *Quelea quelea* (Dallimer 2001).

Calhim S, Birkhead TR (2007) Testes size in birds: assumptions, errors and estimates. Behav Ecol18: 271-275.

Dallimer M (2001) Migration Patterns of the Red-billed Quelea *Quelea quelea* in Southern Africa: Genetics, Morphology and Behaviour [PhD]. Edinburgh, UK: University of Edinburgh.

Darlington RB, Smulders TM (2001) Problems with residual analysis. Anim Behav 62: 599-602

Griffith SC, Owens IPF, Thuman KA (2002) Extra-pair paternity in birds: a review of interspecific variation and adaptive function. Molec Ecol 11: 2195-2212.

Thusius KJ, Peterson KA, Dunn PO, Whittingham LA (2001) Male mask size is correlated with mating success in the common yellowthroat. Anim Behav 62: 435-446.
