# Supplementary figures and images for: Postcopulatory Sexual Selection Is Associated with Reduced Variation in Sperm Morphology
Source: PLoS One. 2007 May 2;2(5):e413. doi: 10.1371/journal.pone.0000413 (PMC1855076; doi:10.1371/journal.pone.0000413)

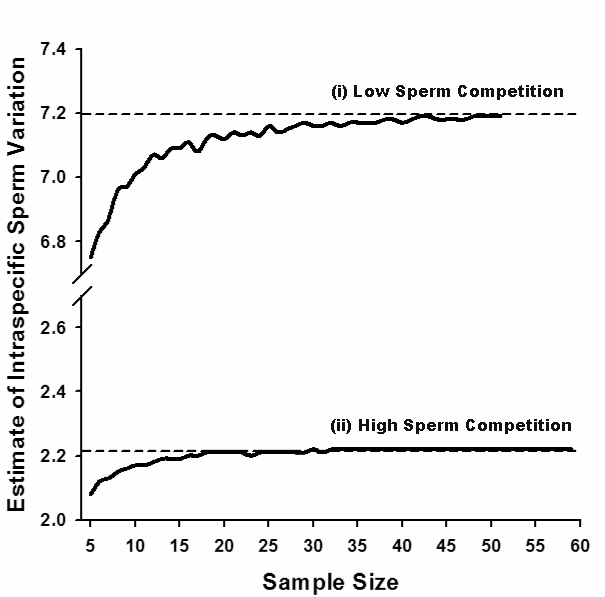

Supplement: Figure S1 — Bootstrapped estimate of the intraspecific coefficient of variation (CV) in sperm tota length (solid lines) against sample size, in species under (i) low or (ii) high sperm competition. The dashed lines correspond to the CV estimate using the complete sample for each species. Note that the n at which the solid lines level off is different in the two cases. (0.04 MB TIF) [file pone.0000413.s002.tif]
